# The Role of Phoneme Discrimination in the Variability of Speech and Language Outcomes Among Children with Hearing Loss

**DOI:** 10.3390/bs15081072

**Published:** 2025-08-06

**Authors:** Kerry A. Walker, Jinal K. Shah, Lauren Alexander, Stacy Stiell, Christine Yoshinaga-Itano, Kristin M. Uhler

**Affiliations:** 1 Department of Physiology and Biophysics, Anschutz Medical Campus, University of Colorado, Aurora, CO 80045, USA; kerry.walker@cuanschutz.edu; 2 Department of Biostatistics and Informatics, Anschutz Medical Campus, University of Colorado, Aurora, CO 80045, USA; jinal.shah@cuanschutz.edu; 3 Department of Audiology, Children’s Hospital Colorado, Aurora, CO 80045, USA; lauren.alexander@childrenscolorado.org; 4 UCHealth Hearing and Balance, University of Colorado Hospital, Aurora, CO 80045, USA; stacy.stiell@uchealth.org; 5 Institute of Cognitive Science, University of Colorado Boulder, Boulder, CO 80309, USA; christie.yoshi@colorado.edu; 6 Department of Physical Medicine and Rehabilitation, Anschutz Medical Campus, University of Colorado, Aurora, CO 80045, USA

**Keywords:** behavioral speech discrimination, hearing loss, early intervention, hearing aids, early auditory skills

## Abstract

This research compares speech discrimination abilities between 17 children who are hard-of-hearing (CHH) and 13 children with normal hearing (CNH), aged 9 to 36 months, using either a conditioned head turn (CHT) or condition play paradigm, for two phoneme pairs /ba-da/ and /sa-ʃa/. As CHH were tested in the aided and unaided conditions, CNH were also tested on each phoneme contrast twice to control for learning effects. When speech discrimination abilities were compared between CHH, with hearing aids (HAs), and CNH, there were no statistical differences observed in performance on stop consonant discrimination, but a significant statistical difference was observed for fricative discrimination performance. Among CHH, significant benefits were observed for /ba-da/ speech discrimination while wearing HAs, compared to the no HA condition. All CHH were early-identified, early amplified, and were enrolled in parent-centered early intervention services. Under these conditions, CHH demonstrated the ability to discriminate speech comparable to CNH. Additionally, repeated testing within 1-month did not result in a change in speech discrimination scores, indicating good test–retest reliability of speech discrimination scores. Finally, this research explored the question of infant/toddler listening fatigue in the behavioral speech discrimination task. The CHT paradigm included returning to a contrast (i.e., /a-i/) previously shown to be easier for both CHH and CNH to discriminate to examine if failure to discriminate /ba-da/ or /sa-ʃa/ was due to listening fatigue or off-task behavior.

## 1. Introduction

As speech discrimination is a foundational auditory skill, the inability to properly categorize speech sounds based on audition alone in childhood can increase the risk of poorer spoken language learning later in life and literacy outcomes (Cantiani et al., 2016; Kuhl et al., 2005, p. 20; 2008; Leppänen et al., 2012; Mueller et al., 2012; Newman et al., 2006; Schaadt et al., 2015; Tsao et al., 2004). In a standard clinical setting, assessments of behavioral speech discrimination among children who are hard of hearing (CHH) or children with normal hearing (CNH) are particularly limited within the first two years of life due to the reliance on language to measure speech discrimination and/or word recognition. Consequently, at prelinguistic ages clinicians instead rely on observational predictors or parental questionnaires to track development. Among older infants and toddlers, condition head turn (CHT) paradigms are utilized to behaviorally measure auditory detection through visual reinforcement audiometry (VRA); however, there is not a common clinical measure used to assess higher level auditory skills necessary for language development, such as speech discrimination. Additionally, there are few reported studies comparing CNH with their aided (tested while wearing hearing aids; HAs) and unaided (tested without their HAs on) CHH peers who are at a greater risk of atypical language development. This increased risk for later language delays highlights the needs for routine behavioral pediatric assessments, beyond auditory detection, to guide intervention and underscores the importance of the presented research.

The use of a CHT paradigm to assess early speech discrimination abilities has been well-documented among CNH cohorts since the 1970s in research investigations (Eilers et al., 1977; Eilers & Oller, 1980; Eisenberg et al., 2007; Martinez et al., 2008; Nozza, 1987). In contrast, there have been fewer reports of behaviorally measured speech discrimination abilities in CHH using HAs or cochlear implants (Govaerts et al., 2006; Martinez et al., 2008; Uhler et al., 2018). Uhler et al. (2018) was among the first researchers to report speech discrimination among CHH using a CHT approach for infants as young as 7 months of age. Uhler’s research reports the findings of infants/toddlers with an average age of 14 months, comparing the speech discrimination of /ba-da/ and /a-i/ among CHH using their HAs and their peers with normal hearing. Notably, infant speech discrimination as measured by CHT has also been linked to both earlier electrophysiologic measures of speech perception, such as acoustic change complex and mismatch negativity (Cone et al., 2022; Uhler et al., 2021a respectively), and later language, including receptive and expressive ability (Tsao et al., 2004; Uhler et al., 2022). These connections across development highlight the potential clinical utility of CHT testing as a measure of speech discrimination to both predict later outcomes and track development. Notably, for speech discrimination to be implemented and used clinically, it is essential that results can be interpreted at an individual level to monitor progress, examine efficacy, and establish normative data in order to compare children with and without amplification. 

Regarding amplification for CHH, Tomblin et al. (2020) reported that the effects of hearing loss on academic outcomes were mediated by the increased audibility provided by HAs. Speech discrimination skills develop not just from earlier acoustic access, facilitated by well-fitted HAs, but more specifically by the quality and quantity of that access. In CHH, the quality of HA fittings can be examined by audibility measures such as speech intelligibility index (SII) and quantity inferred from device datalogging (e.g., average hours of daily hearing aid use), both of which positively relate to better expressive and receptive language outcomes (Tomblin et al., 2020). For example, CHH with well-fit HAs as measured by SII, which measures the audibility of the weighted speech signal (Cole, 2005) in quiet, have a lower risk for language delay than those with poorly fit HAs (SII < 0.61; Wiseman et al., 2023). With regard to datalogging, Tomblin et al. (2015) found that preschool-aged HA users with at least 10 hours of daily hearing aid use had increasing language scores with age supporting the impacts of hearing aids use on language development.

Additionally, HA fittings in CHH depend on the accuracy of thresholds, but the validation of HA fitting is typically demonstrated through behavioral speech discrimination. Unfortunately, speech discrimination tests typically begin at the age of three, after the child has developed spoken language and is able to identify pictures or imitate single words; however, CHT testing with phoneme discrimination provides a prelinguistic tool to assess speech discrimination behaviorally prior to two years of age.

As clinical tools are limited to the language ability of young children, clinicians also encounter patient-level factors that may impact measurement reliability and reduce diagnostic power within this age group. Commonly, auditory testing in young children is discontinued due to listening fatigue or auditory habituation. To mitigate the effects of fatigue and auditory habituation on VRA, clinicians may introduce novel reinforcers and stimuli to enhance response rates (see Visram et al., 2024, for a review). However, it is well-documented that CHH require higher levels of attention and concentration to process auditory information compared to CNH (Bess et al., 2020; Davis et al., 2021; Hornsby et al., 2017). Due to this increased listening effort, CHH may experience greater rates of listening fatigue which can negatively impact test results. Unfortunately, listening effort can be difficult to measure in young children as it is typically ascertained by subjective report, behavioral dual task paradigms, or physiological measures, such as pupillometry or electroencephalography (Shatzer & Russo, 2023). As one can imagine, the implementation of these techniques in real-time, in a pediatric clinical setting, is complex and imperfect for routine VRA. Thus, there is a need to improve existing assessments to simultaneously assess for infant attention, which we refer to as on/off-task orientation, during auditory assessments to improve test reliability.

With regard to infant reliability, a brief review of the literature reveals a handful of studies that have examined the test–retest reliability of behavioral infant speech perception measures among normally hearing children. Cristia et al. (2016) reported on the test–retest reliability of 12 studies completed by three laboratories using behavioral speech perception assessment techniques, central fixation or head turn preference, on infants aged 5 to 12 months. Analysis between repeated measures revealed a wide range of correlation coefficients ranging from −0.44 to +0.87, whereas only three studies had positive relationships (Cristia et al., 2016). Most relevant to our protocol is a study from Nozza et al. (1991), which examined the reliability of infant thresholds for speech discrimination in noise to a consonant contrast, /ba-ga/. Between two test sessions, individual infant thresholds, as measured using a CHT procedure, varied by less than 10 dB for 87.5% of the 16 tested infants which is comparable to the variability of adult audiometric testing (Stuart et al., 1991). This study was completed prior to the establishment of universal newborn hearing screenings and has not been replicated in CHH with early auditory access. Examples such as these motivate our reasoning to also employ easier contrast trials (e.g., /a-i/) to assess for non-auditory influences, such as fatigue or attention, for CHT testing to better understand variability in speech discrimination outcomes.

In the following study, children with and without hearing loss are assessed using a CHT and conditioned play paradigm to two consonant-vowel contrasts. As Uhler et al. (2018, 2021b) reported that between 75 and 95% of infants could discriminate the vowel contrast, /a-i/, our study utilized the ‘easier’ vowel contrast to assess on/off-task orientation (i.e., capacity to continue attending to the task “on/off-task”) during assessments and reduce variability due to non-sensory factors.

Study objectives:

1. Compare discrimination abilities between CHH (aided, with both HAs on and unaided, without HAs) and CNH.
2. Utilize a vowel contrast to assess infants on/off-task orientation during speech discrimination testing.
3. Determine if speech discrimination abilities improved upon repeat testing following successful on-task assessment.

## 2. Materials and Methods

The local Institutional Review Board approved this project and was registered on clinicaltrials.gov (NCT05653999). Consent was obtained from parents/guardians before beginning the research protocol. Parents were provided with compensation for their child’s participation. Participant compensation was paid in cash or gift cards at a rate of either $15 per hour or $40 for each study visit. Compensation was increased following the initiation of the study to enhance participant retention.

### 2.1. Participant Information

Criteria for inclusion were (a) age between 6 months and 36 months at the time of enrollment, (b) English as the reported primary spoken home language, and (c) demonstrated ability to complete a conditioned response via VRA or conditioned play audiometry. For CNH, additional criteria included (d) passed newborn hearing screening and otoacoustic emissions (OAE) testing following enrollment. For CHH, additional criteria included (e) diagnosis of bilateral sensorineural hearing loss ranging from mild to severe, (f) full-time use of amplification as reported by managing audiologist, and (g) current enrollment in early intervention. 

Criteria for exclusion were (a) gestational age earlier than 35 weeks, (b) abnormal tympanometry bilaterally on the day of testing, (c) concerns of secondary disabilities per parent report or medical record review, (d) auditory neuropathy diagnosis, and for CNH, (e) concerns for hearing loss, and (f) failure to condition to any contrast. 

A total of 36 participants were enrolled in the study (20 CHH and 16 CNH). Following enrollment, six participants were excluded. Two participants, one in each cohort, were never able to condition to the task. A single CHH participant was also excluded due to inability to condition to the task and for concerns of secondary disabilities per parent report following enrollment. Three additional participants, two CHH and one CNH, were withdrawn and did not contribute data due to bilateral abnormal middle ear function as assessed through tympanometry and/or distortion product otoacoustic emissions during the test session. Of the remaining 30 participants (17 CHH and 13 CNH), the average age of enrollment was 22.3 months and 19.2 months for the CHH and CNH cohorts, respectively.

### 2.2. Demographics

Prior to exclusions, the 36 enrolled participants were matched in terms of age and sex across cohorts. Of the remaining 30 participants, 6 participants had pressure equalization tubes with an average age of placement at 17.5 months. All parents of participants confirmed that gestational age was greater than 35 weeks prior to enrolling in the study. The exact gestational age was known for 29 participants with an average of 39 weeks. Between both cohorts, majority of participants identified as white (*n* = 29) and non-Hispanic/Latino (*n* = 23), see Table 1 below for detailed demographic information.

### 2.3. Demographic Information for CHH

The age of identification of hearing loss ranged from 8 to 96 days (average 35.4 days). CHH enrolled in the study had bilateral mild to severe hearing loss based on their pure tone average. The 17 CHH participants were diagnosed with either mild (*n* = 8) or moderate (*n* = 9) sensorineural hearing loss. Following diagnosis, children were fit with bilateral, behind-the-ear air conduction HAs, coupled to a soft fully occluding custom earmold, and programmed using Desired Sensation Level (DSL) v5.0 (Scollie et al., 2005). Children each received individualized care from a managing audiologist at a local Colorado audiology center following current best practices and recommended fitting guidelines (American Academy of Audiology, 2013; Bagatto et al., 2023). The children in this sample were fit on average at 2.6 months of age (range 1.45–5.13 months). All participants utilized either HA manufacturer A (*n* = 11) or B (*n* = 6) devices. For children who were fit with manufacturer A HAs, they wore their personal amplification during testing. For testing, all CHH utilized manufacturer A HAs for laboratory testing. HA selection was determined by matching their current power level programmed to their thresholds using DSL, matched to speech mapping targets. 

Daily HA device use was obtained from personal HAs, using datalogging, during the test session. The average datalogging observed was 7.2 h of daily use (range 0.12–16 h). These measurements were conducted with the CHH’s personal HAs.

HA verification data was used from the participant’s medical records if values were recently obtained (within one month) by their managing audiologist using measured RECDs and if the participant had not received new earmolds since the time of their research visit. If the child had received new earmolds since their most recent standard of care visit, or if average RECDs were previously used, the study team completed HA verification during a study visit. Measured RECDs were attempted for all participants and average RECDs (*n* = 3) were used if measured could not be obtained. Aided SII, unaided SII, and aided RMSE were recorded for soft (50/55 dB), average (60/65 dB), and loud (70/75 dB) inputs using DSL prescriptive speech mapping targets. 

See Appendix A Table A1 for variables specific to CHH.

### 2.4. Stimuli

For this protocol, three speech contrasts were used, /ba-da/, /sa-ʃa/, and /a-i/. Speech discrimination ability to consonant contrasts was the primary outcome of interest, and the vowel contrast was inserted into the protocol, as needed, to probe the infant’s on/off-task orientation. Contrasts were selected based on previous work completed by the Uhler group (Uhler et al., 2018, 2021b). Vowel contrast /a-i/ and consonant contrast /ba-da/ are previously detailed in (Uhler et al., 2011). All speech tokens were 500 msec in duration. Vowel duration was the full 500 msec and consonant vowel tokens consisted of a 100 msec consonant (i.e., /b/, /d/, /s/, /ʃ/) followed by a 400 msec vowel interval. During testing, speech tokens were presented in groups of three (i.e., /ba-ba-ba/) and a 1200 msec interstimulus interval. The presentation order was randomized and, within each stimulus pair, either contrast could serve as the background token. The other speech sound served as the target token (e.g., background: /ba-ba-ba/, target: /da-da-da/). The member of the pair serving as the target token was counterbalanced across participants. All stimuli were presented at 60 dB SPL and were calibrated in the sound field using an A-weighted scale. Table 2 lists the formant frequencies for each stimulus.

### 2.5. Testing Protocol

One to five sessions were required to complete the speech discrimination protocol. The first session consisted of the case history (information related to the child’s general health, development, years of education of the child’s mother, and information related to HA use/care), screening for abnormal middle ear function, HA measurements (data logging and speech mapping), and the discrimination task. Subsequent sessions were scheduled if the full CHT protocol could not be completed in the first session due to failure to condition, inability to reliably continue the task due to fussiness or fatigue, observed abnormal middle ear function, or when halted by the testing protocol following poor performance on the /a-i/ assessment. 

Two speech contrasts were assessed (/ba-da/ and /sa-ʃa/) during speech discrimination testing and a third speech contrast (/a-i/) was used as needed to assess if the child was still on-task. All contrasts were presented at 60 dB SPL. For children in the CHH cohort, each contrast was assessed in the aided and unaided conditions. To control for learning effects, CNH were tested twice on each contrast. When /a-i/ was used to assess on/off-task orientation, this was completed in the aided condition for the CHH cohort. 

Testing was completed in a sound treated room. Caregivers accompanied children into the sound booth for the discrimination assessment. Prior to entering the room, the background token was playing continuously and continued while they entered the room. Children were either seated on the caregiver’s lap or in a highchair in the center of the room to minimize distractions or task fatigue. Regardless of positioning, the distance between the child’s head and the speaker was the same. The speaker and visual reinforcement video screen were 90° to the right of the child’s midline. An assistant was positioned in front of the child, slightly to the left, to center their gaze. During testing, the caretaker and the assistant listened to music and masking noise through headphones to prevent them from hearing the sounds presented and accidentally cueing the child to a contrast change. The evaluator observed through a window in a separate room outside the sound booth and was also blinded to the stimulus while testing. 

The discrimination testing protocol comprised two phases: conditioning and testing. In the conditioning phase, only change trials were conducted so infants could learn the association between a change in sound and the reinforcer. Conditioning was completed in the aided condition for the CHH cohort, regardless of whether the randomized first contrast was tested aided or unaided. During conditioning, an intensity cue was used where the target token was presented at a louder level than the background token (+6 dB SPL) to alert the child to the stimulus change. Initially, the reinforcer was automatically activated after two target tokens were presented. After the child made two independent, consecutive head turns before the end of the first two presentations of the target token, the intensity cue was removed. Trials were initiated by a button press once the evaluator determined that the child’s attention was directed toward the midline. As needed (*n* = 16), an intensity cue was used to train the child for the second contrast. The testing phase proceeded as described above. 

For older children for whom the CHT task was too rudimentary for the child’s development, a conditioned play-based task or a combination of a conditioned play task and CHT was utilized. The conditioning and testing phases were identical between task types; however, in the conditioned play-based task the child was conditioned to a task such as placing a toy in a bucket upon hearing a change in the stimulus instead of completing a head turn. 

When utilizing the CHT (*n* = 27), computer software determined trial-type presentation, with either 7 or 8 of the 15 trials being a change or no-change trial as randomly determined by the computer; the evaluator was blind to trial type. If the trial was a no-change trial, the background token was presented. If the trial was a change trial, the target token was presented. At the end of each trial, the background token continued. The evaluator indicated when the child executed a head turn toward the speaker by button press. The CHT software determined if the child’s head turn was a correct response to a change trial or a false positive to a no-change trial. Correct responses were rewarded by the automatic presentation of a visual reinforcer, an animated video. Performance on the task was quantified using d-prime (Macmillan & Creelman, 2005). If the child achieved a d′ ≥ 1.2, then testing was complete for that contrast (Uhler et al., 2022).

When utilizing the conditioned play task (*n* = 3), computer software determined trial-type presentation, with half of either 8 or 12 trials being a change or no-change trial as randomly determined by the computer. During the session, scoring was a binomial score and if the child scored greater than 0.50, then testing was complete for that contrast. The d′ score was then calculated offline following the session to be compared with CHT scores. If the child could not complete the conditioning phase, could not reliably complete the testing phase (i.e., fussiness, irritable), or meet criterion (d′ ≥ 1.2) they were categorized as unable to discriminate that contrast.

**Figure 1:**
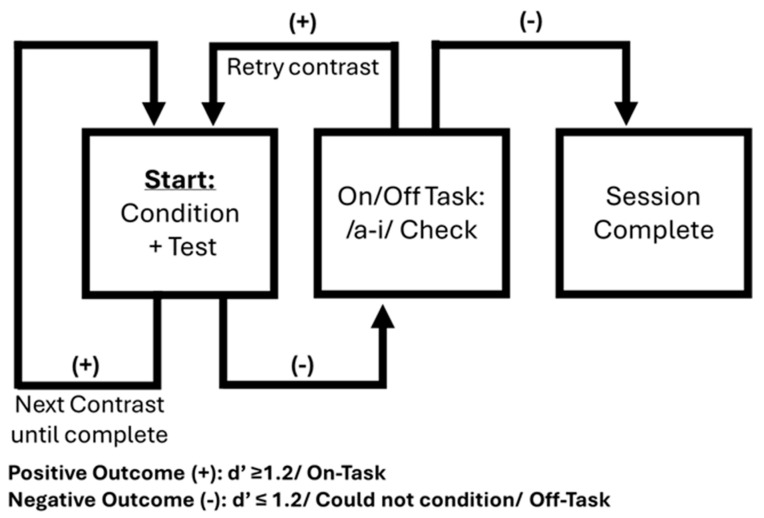
Speech discrimination testing protocol. This flow chart depicts the testing protocol for CNH and CHH. Ordering of contrasts was predetermined and randomized across subjects to control for order effects. CHH always completed the conditioning phase while aided and HAs were removed prior to testing unaided conditions. CHH also completed on/off task assessments while aided.

Figure 1 demonstrates the testing protocol used. If the child reached criterion on the first contrast, the tester would then move to conditioning and subsequent testing of the second contrast. This would continue until all contrasts were completed or criterion was not reached on a contrast. The vowel /a-i/ contrast was used to assess on/off-task orientation only if criterion was not met on a contrast in the best listening condition (aided for the CHH cohort). If the child had previously reached criterion on a contrast, the tester looked for two independent consecutive responses for the /a-i/ contrast and determined the child as on-task before continuing with the protocol. If the child had not previously met the criterion on a contrast, a full test of /a-i/ was completed, and the protocol was resumed if the child met criterion (d′ ≥ 1.2) and was therefore considered to be on-task. If the child failed to demonstrate two independent consecutive responses or did not meet criteria for a full test of /a-i/, they were determined to be off-task, that session was discontinued and a subsequent session was scheduled. Each contrast (/ba-da/ and /sa-ʃa/) in the best listening condition could be tested up to two times in a single session should criterion not be met upon the first test.

#### Tympanometry and OAE

Screening for abnormal middle ear function consisted of tympanometry for the CHH cohort, and tympanometry and DPOAEs for the CNH cohort. If abnormal middle ear function was identified in either ear, the session was discontinued. Abnormal middle ear function for the CHH was classified as a flat type B tympanogram with normal ear canal volume. The abnormal middle ear function for the CNH cohort was classified as a flat type B tympanogram with normal ear canal volume and a refer on the DPOAE screening (pass was present DPOAE for at least three of the four frequencies tested).

### 2.6. Statistical Analysis

To complete our first objective, ‘Compare CNH and CHH speech discrimination abilities’, the Wilcoxon rank-sum test and paired *t*-tests were performed. This comparison allowed us to examine the effect of hearing status on speech discrimination using the Wilcoxon rank-sum test and the effect of aided vs. unaided status for CHH using paired *t*-test. The Wilcoxon rank-sum test was chosen because the distribution of d′ scores in the CNH and CHH groups was non-normal. In contrast, the paired *t*-test was used for the CHH comparisons because the distribution of within-subject differences was approximately normal. When examining cohort differences, if a child had multiple scores for a given contrast, the best d′ score was entered into the Wilcoxon rank-sum test. When examining the effects of amplification for CHH, a paired *t*-test was used only for infants with 2 consecutive d′ scores for a given contrast. In addition to analysis using d′ as a continuous variable, individual scores were also categorized based on our criterion of a d′ ≥ 1.2, indicating discrimination of that contrast pair (Uhler et al., 2022).

Additionally, to better understand variability within CHH, multiple Pearson correlations were run using speech discrimination scores and HA-related variables. This included age at ID, age at fit, aided SII, RMSE, and datalogging. The child’s best single score for each contrast was used for this analysis.

Next, to ‘utilize a vowel contrast to assess infant on/off-task orientation during speech discrimination testing,’ child performance was coded as either, (1) on-task or (2) off-task following /a-i/ assessment. Additionally, by comparing speech discrimination scores preceding and following /a-i/ assessment, child performance was further coded for analysis based on differences between score 1 and score 2. Children that were deemed on-task were further coded as (1.1) no change in score (i.e., Score 1 = Score 2), (1.2) improvement to score (i.e., Score 1 did not meet criterion then Score 2 met criterion) or (1.3) worsened score (i.e., Score 1 met criterion then Score 2 did not meet criterion). Off-task infants were also further categorized into (2.1) no change in score (i.e., Score 1 = Score 2) or (2.2) fatigue (i.e., Score 1 did not meet the criterion, then Score 2 met the criterion).

Descriptive summaries of performance are provided by frequency for each respective cohort, and Fisher’s Exact test was performed to investigate on/off task observation among CHH and CNH. Due to the increased listening effort required by CHH, we hypothesize that more CHH will exhibit off-task characteristics. Additionally, we hypothesize that if a child did not reach criterion upon first assessment of the consonant-vowel contrast (/ba-da/ or /sa-ʃa/), but demonstrated that they were on-task, their speech discrimination score upon repeating the consonant-vowel contrast should improve or stay the same. Alternatively, if their scores remained the same or poorer it could be used to differentiate non-sensory factors and discrimination abilities. Notably if a subject initially met criterion the target contrast, based on our testing protocol, that subject was not assessed for on- and off-task behavior before moving to the next contrast and, therefore, not all subjects were assessed for on/off-task behavior.

To investigate our final objective, ‘Determine if speech discrimination abilities improve upon repeated testing’, a Wilcoxon signed ranks test was performed. This allowed us to compare speech discrimination scores of the consonant-vowel contrasts when repeated within and across testing sessions. Lastly, a Wilcoxon rank-sum test was used to compare the differences in scores between repeated testing across cohorts.

Results were considered statistically significant at an alpha level of 0.05. All analyses were performed using R software v4.4.1 (R Core Team, 2021). 

## 3. Results

### 3.1. Speech Discrimination Outcomes

**Figure 2:**
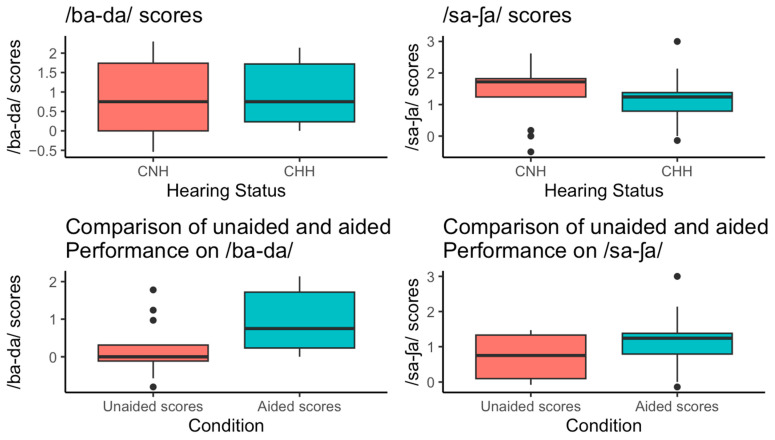
Distribution of speech discrimination scores across groups. Figure displays the distribution of /ba-da/ and /sa-ʃa/ scores across hearing groups and conditions. In the top panels, /ba-da/ and /sa-ʃa/ scores are shown for CNH and CHH. Both groups demonstrated similar distributions in /ba-da/ scores, while /sa-ʃa/ scores were higher and less variable among CNH compared to CHH. The bottom panels focus on CHH participants, comparing unaided and aided conditions. For both /ba-da/ and /sa-ʃa/ scores, aided conditions tended to show higher median scores compared to unaided conditions, suggesting potential performance improvement with amplification.

Sixteen aided CHH and 13 CNH completed the /ba-da/ testing protocol. Seventeen aided CHH and 13 CNH completed the /sa-ʃa/ testing protocol. The distribution of scores shown in Figure 2. Mean /ba-da/ scores were similar between aided CHH (0.91 ± 0.76) and CNH (0.85 ± 1.00), with a median of 0.75 in both groups. In contrast, /sa-ʃa/ scores were slightly lower in aided CHH (1.13 ± 0.73) compared to CNH (1.45 ± 0.97), with a median of 1.24 and 1.72, respectively. Criterion-based categorizations of these scores showed 38% met criteria for /ba-da/ in both groups, while 53% of aided CHH and 77% of CNH met the criterion for /sa-ʃa/. Unaided /ba-da/ and /sa-ʃa/ scores for CHH participants had means of d′ = 0.21 (±0.74) and d′ = 0.73 (±0.60), respectively.

See Appendix A Table A2 and Table A3 for participant details regarding testing for each contrast.

### 3.2. Comparison of Aided vs. Unaided Scores for CHH

There was a significant difference between /ba-da/ scores for aided CHH compared to unaided CHH (t (df = 11) = 2.69, *p* = 0.02). Specifically, mean aided /ba-da/ scores were 0.93 (95% CI [0.17, 1.68]) higher compared to mean unaided /ba-da/ scores.

However, there was not a significant difference between aided and unaided CHH /sa-ʃa/ scores (t (df = 12) = 1.69, *p* = 0.12). Mean aided /sa-ʃa/ scores were 0.35 (95% CI [−0.1, 0.79]) higher compared to mean unaided /sa-ʃa/ scores. The difference remained insignificant when we removed the one outlier with very high aided /sa-ʃa/ scores (t (df = 11) = 1.27, *p* = 0.23).

### 3.3. Comparison of Scores for Aided CHH vs. Scores for CNH

No significant group differences were observed when comparing aided CHH and CNH for performance on /ba-da/ (W = 98.5, *p* = 0.81). When comparing performance between groups for the /sa-ʃa/ contrast, scores differed significantly between CNH and aided CHH (after removing one outlier) as indicated by Wilcoxon rank-sum test (W = 151, *p* = 0.04). Specifically, aided CHH had a lower median of 1.19 when excluding the outlier, compared to a higher median of 1.24 when the outlier was included, and a median of 1.72 for CNH participants. Note, following visualization of the distribution of scores for CHH and using boxplots, the /sa-ʃa/ outlier was removed due to its value being 1.5 times the interquartile range of the third quartile.

### 3.4. HA and Participant Factors

Multiple Pearson correlations revealed no significant relationships between CHH factors (age at ID, age at fit, PTA, aided SII, RMSE, Datalogging) and speech discrimination scores (all *p* > 0.05). Comparisons within CHH sorted by a child’s personal hearing aid manufacturer revealed no significant differences in performance on either contrast (*t*-test, *p* > 0.05).

### 3.5. On/Off-Task Orientation

Among 17 infants (8 CHH, 9 CNH) with /ba-da/ scores where /a-i/ was used to check on/off-task behavior, the distribution of categories differed slightly between hearing groups (Table 3). Participants included in these analyses did not reach criterion on the respective speech sound contrasts. 

Among 11 children (6 CNH, 5 CHH) with /sa-ʃa/ scores for the /a-i/, most were categorized as on-task, with patterns differing between hearing groups (Table 4). Off-task behavior was uncommon; off-task with no change was noted in 18% of the overall sample, and no children showed off-task fatigue. 

### 3.6. Repeated Testing Outcomes

Based on the testing protocol, 17 children (9 CNH and 8 CHH) completed repeated testing for /ba-da/. Overall, in the entire sample, the mean and median differences between first and second /ba-da/ scores were 0.37 and 0, respectively. There were no differences observed between the second and first /ba-da/ score (V = 21, *p* = 0.17) for the whole sample. Furthermore, the difference in second and first score between CNH and CHH group were also not significantly different (W = 44.5, *p* = 0.72).

For /sa-ʃa/, 14 children (7 CNH and 7 CHH) completed repeated testing. In this sample, the mean and median difference between first and second /sa-ʃa/ scores were 0.41 and 0.38, respectively. However, the difference was found to be not significant between second and first /sa-ʃa/ score (V = 15, *p* = 0.07). Furthermore, the difference between CHH and CNH groups were also not significantly different (W = 32.5, *p* = 0.31).

## 4. Discussion

The main goal of this study was to compare speech discrimination outcomes of CNH and CHH as measured by a CHT or condition play paradigm. Additionally, a secondary aim was to examine the utility of a vowel contrast to determine on/off-task orientation as individual child variability can influence test results and impact clinical decision making. Finally, when applicable, we examined if child speech discrimination scores improved upon repeated testing. These results also demonstrate the ability to capture individual speech discrimination ability at an average age of 20 months using a clinically viable testing method.

### 4.1. Speech Discrimination

While this research protocol allowed us to observe novel findings, it also replicated previous findings from our lab work utilizing a CHT paradigm in CHH and CNH. Primarily, this work is the first to demonstrate effectiveness, in aided versus unaided speech discrimination abilities, of early identified and early amplified very young children. When collapsing across groups, 33/35 of the enrolled typically developing children could be conditioned to the speech discrimination task and, of the 30 included for analyses, 83% could discriminate at least one contrast (i.e., /a-i/, /ba-da/, or /sa-ʃa/). With respect to the /ba-da/ contrast, 38% of both CHH and CNH participants could meet criterion indicating successful discrimination of that vowel-consonant contrast. This demonstrates that through Universal Newborn Hearing Screening/Early Hearing Detection and Intervention (UNHS/EHDI) programs, well timed early intervention and amplification, CHH performed similarly to CNH age and sex matched peers on behavioral assessments of speech discrimination. Next, this work also successfully replicates previous research from our lab on CHH and CNH speech and discrimination ability. Uhler et al. (2021b) used a similar CHT paradigm at three intensities, 50, 60, and 70 dB, and overall found that 58% of aided CHH and 51% of CNH could discriminate /ba-da/. However, a difference with our current study is that we tested infants at a single intensity, 60 dB SPL. When examining the percentage of infants from the 2021 study that could successfully discriminate /ba-da/ at 50 and 60 dB, assuming that criterion can be met at a higher intensity if previously achieved at lower intensity (McArdle & Hnath-Chisolm, 2009), we see that 34% of CHH and 38% of CNH successfully discriminated /ba-da/. This is very similar to the percentages for the current study’s cohort of CHH and identical for CNH. Findings also suggest that testing at a greater intensity, such as 70 dB, may result in more children successfully discriminating /ba-da/.

With respect to the /sa-ʃa/ contrast, there was a significant difference between groups such that CNH performed better than CHH whereas, 77% of CNH met criterion versus 53% of CHH. This difference in performance may be due to increased perceptual difficulties commonly experienced by CHH, specifically for mid-to-high frequency sounds. Research from Stelmachowicz et al. (2002) investigated the detection of /s/ and /z/ morphemes in CHH and CNH revealed that CNH can correctly detect these inflectional morphemes above chance levels with increasing rates up to >90% from 3 to 5 years of age. However, when compared to older CHH aged 5 to 13 years, their team observed considerable variability in CHH speech perception, highlighting the persistent perceptual difficulties experienced by CHH and the importance of access to mid and high frequencies to perceive phonemes denoting plurality. Of note, it is likely that this older CHH cohort may not have been early identified through a UNHS program and warrants replication. With regard to our study, we observed that around 50% of early identified, and younger children, could successfully discriminate /s/; however, a significant difference between CHH and CNH was also observed. 

Future research could also explore the impact of frequency lowering on the speech discrimination of fricatives among infants and toddlers who use HAs. While this has been studied among older children (Auriemmo et al., 2020; Glista et al., 2017), research at younger ages at which foundational auditory skills are developed is necessary, as the implementation of frequency lowering hearing aid processing varies across manufacturers, and patient-specific factors must be considered when using this feature (Glista & Scollie, 2018). In an exploratory analysis within our CHH cohort, we examined potential differences in speech discrimination between users of different HA manufacturers and neither continuous nor categorical comparisons revealed significant differences (*p* > 0.05) or consistent trends between groups. This absence of group effects may be due to small sample sizes. Additionally, all CHH were tested using hearing aid manufacturer A devices, which may have influenced outcomes. Results might differ if participants used their personal HAs across testing sessions, given the variability in manufacturer-specific signal processing.

Interestingly, a larger proportion of both groups met criterion on the /sa-ʃa/ contrast compared to /ba-da/, suggesting that the difference in performance may also be stimulus-dependent. Notably, while the /ba-da/ stimuli were normalized for fundamental frequency (F0), the /sa-ʃa/ stimuli were not. This decision was based on pilot testing, which revealed that even CNH participants were unable to discriminate the /sa-ʃa/ contrast when F0 was normalized. Prior studies have emphasized the role of F0 access in speech discrimination, particularly in challenging listening conditions (Oxenham, 2008). Furthermore, F0 normalization introduces relatively small (~50 Hz) differences in the /ba-da/ formant transitions, whereas the /sa-ʃa/ contrast features larger spectral differences of approximately 200 Hz (see Table 2).

Another point of interest was whether characteristics specific to CHH influence performance on speech discrimination. As previous research has shown relationships between auditory access facilitated by HAs (Farquharson et al., 2022; Uhler et al., 2018, 2021b) and speech perception. From (Uhler et al., 2018), we also examined relationships between CHH specific variables (i.e., aided SII) and speech discrimination outcomes. However, in the current study, no significant relationships were observed between CHH variables and speech discrimination performance. 

### 4.2. On/Off-Task Orientation

In a subset of our subjects, those who did not reach criterion on the speech discrimination task were assessed for on/off-task orientation using a vowel contrast, /a-i/. A total of 17 infants (9 CNH, 8 CHH) were assessed during /ba-da/ testing, and 11 children (6 CNH, 5 CHH) were assessed during /sa-ʃa/ testing. It is important to note that these individuals were who performed poorly on the discrimination task and that children who reached criterion were not assessed for on/off-task orientation. Additionally, /a-i/ may have been used between two differing contrasts; however, this analysis only includes instances across two consecutive scores of the same contrast. Our study team hypothesized that more CHH would exhibit off-task behavior due to the increased concentration and listening effort required for CHH’s auditory processing compared to CNH. Additionally, we hypothesized that for children who demonstrated that they were on-task, their speech perception score upon repeating the consonant-vowel contrast should improve or stay the same.

Overall, the majority of infants were on-task, meaning that they met criterion or head-turn check requirements for /a-i/ testing. With regard to on/off-task orientation, for /ba-da/, the categorization of on/off task orientation was nearly identical between cohorts with 12 infants coded as on-task (6 CNH, 6 CHH) and 5 as off-task (3 CNH, 2 CHH). For /sa-ʃa/, a similar breakdown was observed for on- versus off-task with 9 on-task (5 CNH, 4 CHH) and 2 off-task (1 CNH, 1 CHH). From these observations, we did not see a larger proportion of CHH exhibiting off-task behavior as expected from our hypothesis. One possible explanation is that CHH may have been more familiar with the CHT paradigm due to routine exposure through VRA in clinical settings. Our inclusion criteria required children to demonstrate the ability to complete conditioned response testing via either VRA or Conditioned Play Audiometry (CPA). As a result, many of the CHH in this small cohort were likely already accustomed to CHT-like paradigms. In contrast, CNH may have been less familiar with the task, which could have contributed to a greater likelihood of off-task behavior. Although one more CNH child was observed to be off-task compared to CHH, this difference was not significant. While our current dataset does not allow us to test this directly, it is possible that CNH, unlike CHH, had more resources to allocate to learn the novel testing method, as they may have required less effort to process the auditory stimuli. An additional point of interest observed from our dataset shows that more CNH improved following /a-i/ testing while a majority of on-task CHH had no change in scores between score 1 and 2. This finding follows our secondary hypothesis that if children were deemed on-task, then we would expect improvements or stable scores across test trials. This may again speak to the additional capacity for learning by CNH, as this could be interpreted as CNH learning the task and improving speech perception with practice. For CHH, their ability was accurately ascertained through initial testing and was stable if they remained on-task. Upon further testing, this approach could be used to differentiate when a child could truly not discriminate the speech sound contrast, rather than other sensory factors influencing the outcomes. Although interesting, statistical analysis did not reveal any differences between cohorts indicating similar performance. 

### 4.3. Repeated Testing Outcomes

Finally, as children may have been tested on a contrast multiple times based on our protocol, our study team was able to assess changes across assessments in a subset of CNH and CHH. Specifically, 17 (9 CNH and 8 CHH) and 14 children (7 CNH and 7 CHH) completed repeated testing for /ba-da/ and /sa-ʃa/, respectively. It is again important to note that some CHH may not have completed repeated testing if they reached criterion on an initial contrast and that this current study does not formally assess test–retest reliability. Statistical analysis showed no significant change in mean or median scores between assessments, and the magnitude of score differences was similar across cohorts suggesting no differences in performance over time. In contrast, Uhler et al. (2011) found that speech discrimination performance in children with cochlear implants, measured prior to implantation, at activation, and at 1, 2, and 3 months post-activation, was comparable to that of age- and sex-matched peers with normal hearing by three months post-implantation. These findings suggest that meaningful improvements in discrimination performance are more likely to result from changes in auditory access (i.e., cochlear implantation) rather than repeated exposure to the testing paradigm. Clinically, this suggests that speech discrimination scores are unlikely to change within a month in the absence of a change in intervention. Future research could examine changes in discrimination performance longitudinally to better highlight windows of stable performance or potential change for CHH and CNH. When compared to the 12 studies summarized by Cristia et al. (2016), their group observed high rates of variability across several measures of speech perception in the younger cohort of children across a smaller window of test–retest. Similarly to Cristia’s findings, pediatric speech discrimination does contain within-subject variability which highlights the need for unbiased statistical tools, such as signal detection theory, and consensus-driven test parameters and scoring techniques to enhance generalization across studies and timepoints. 

### 4.4. Clinical Implications and Limitations

Broadly, this current study utilizes a testing procedure that can be applied in a clinical setting to demonstrate amplification benefit or validate amplification fittings in children as young as 6 months of age. As a strength, a single condition can also be completed in 5 to 6 min (Uhler et al., 2022) further highlighting feasibility of fitting this measure into a clinical evaluation and prompting research regarding the clinical implementation of such procedures. Additionally, the use of an easier /a-i/ token can be employed to assess for child on-task orientation in a similar fashion to the usage of suprathreshold pure tones to refamiliarize difficult to test adult patients during audiometry (ASHA, 2005).

However, as this study contributes novel findings regarding the assessment of speech discrimination behaviorally in young children, this study does also have limitations. Primarily, it should be taken into consideration that these findings are mainly generalizable to children who were early identified, early amplified, typically developing, and receive audiologic care in an urban setting. These results for CHH may differ based on the type of care and intervention received. Additionally, as we expected to observe influences in speech discrimination ability based on auditory access, no significant relationships were observed. This may be due to the sample size, which limits statistical power, or to the lack of variability in HA fitting such that all CHH had well-fitted hearing aids. The average recorded RMSE for inputs of 60 dB was 2.74, well within the recommended fitting guideline of 5 dB (Wiseman et al., 2024), indicating consistently high-quality HA fittings. In a larger or more heterogeneous sample, such as that examined by Uhler et al. (2021b), significant associations may be more likely to emerge as we previously found a positive relationship between aided SII and speech discrimination. 

Similarly, a small sample size may have inhibited our ability to observe statistical differences across categorical comparisons of on/off-task behavior. Based on our protocol, children were only assessed for on/off-task assessment if they did not reach criterion on the initial contrast which limited our sample size. Future research will include assessment of children who meet criterion to allow for a more robust comparison of performance within and across cohorts, as well as, across timepoints.

## 5. Conclusions

This research is the first to compare the behavioral speech discrimination of CHH and CNH, using a CHT technique or conditioned play technique, with and without amplification at the average age of 20 months. With amplification, CHH had similar speech discrimination performance to CNH, highlighting the importance of early identification, early amplification and early therapeutic intervention for CHH. However, group differences emerged for the discrimination of fricatives but not for stop consonants. Additionally, the benefit of amplification was observed for CHH’s ability to discriminate /ba-da/. Repeated CHT testing within 4 weeks did not significantly change speech discrimination scores, further highlighting the utility of CHT as a reliable assessment of speech discrimination ability in CHH. Finally, the use of an easier vowel contrast, such as /a-i/ can help probe external patient factors, such as attention, which may influence reliability of outcomes and future clinical decision making.

## Figures and Tables

**Table 1:** Sample characteristics stratified by hearing group.

	Overall (*n* = 30)	CHH (*n* = 17)	CNH (*n* = 13)
**Sex**			
Male	14 (47%)	7 (41%)	7 (54%)
Female	16 (53%)	10 (59%)	6 (46%)
**Race**			
White	28 (93%)	16 (94%)	12 (92%)
Black	0 (0%)	0 (0%)	0 (0%)
Asian	1 (3%)	1 (6%)	0 (0%)
Native American	1 (3%)	0 (0%)	1 (8%)
**Ethnicity**			
Hispanic/Latino	7 (26%)	2 (12%)	5 (38%)
Non-Hispanic/Latino	23 (74%)	15 (88%)	8 (62%)
**Maternal Level of Education**			
High School	2 (7%)	2 (12%)	0 (0%)
Vocational/Some College	3 (10%)	1 (6%)	2 (15%)
Associate’s	2 (7%)	2 (12%)	0 (0%)
Bachelor’s	9 (30%)	6 (35%)	3 (23%)
Master’s	6 (20%)	5 (29%)	1 (8%)
Doctoral	8 (27%)	1 (6%)	7 (54%)

Note. TAhis table summarizes participant demographic characteristics for CNH and CHH included in the analysis following exclusions.

**Table 2:** Stimuli spectral characteristics.

Stimulus	1st Formant	2nd Formant	3rd Formant	4th Formant
/a/	629.7	1227.6	2850.7	3915.7
/i/	336.5	2897.7	3781.2	4618.4
/ba/	841.6	1341.0	2866.2	3161.6
/da/	857.8	1288.5	2904.3	3084.0
/sa/	727.4	1574.4	2827.9	3643.1
/ʃa/	517.7	1920.6	2791.7	3889.3

Note. Average frequencies for formants 1 through 4 of each stimulus, reported in Hertz. Of note, the fundamental frequency (F0) was normalized for /ba-da/ and /a-i/ but not for /sa-ʃa/ due to difficulties assessing children during pilot data collection.

**Table 3:** Categorization of on/off-task orientation following /ba-da/.

	Overall (*n* = 17)	CNH (*n* = 9)	CHH (*n* = 8)	*p*-Value
**/a-i/ categories for /ba-da/**				>0.9
**On-Task**				
On-Task: No Change	6 (35%)	3 (33%)	3 (38%)	
On-Task: Improved	6 (35%)	3 (33%)	3 (38%)	
On-Task: Worsened	0 (0%)	0 (0%)	0 (0%)	
**Off-Task**				
Off-Task: No Change	5 (29%)	3 (33%)	2 (25%)	
Off-Task: Fatigue	0 (0%)	0 (0%)	0 (0%)	

Note. This table displays the on/off-task categorization of participants that completed /a-i/ assessments during /ba-da/ testing. Fisher’s exact test showed no statistically significant difference between groups (*p* > 0.9).

**Table 4:** Categorization of on/off-task orientation following /sa-ʃa/.

	Overall (*n* = 11)	CNH (*n* = 6)	CHH (*n* = 5)	*p*-Value
**/a-i/ categories for /sa-ʃa/**				0.11
**On-Task**				
On-Task: No Change	4 (36%)	1 (17%)	3 (60%)	
On-Task: Improved	4 (36%)	4 (67%)	0 (0%)	
On-Task: Worsened	1 (9.1%)	0 (0%)	1 (20%)	
**Off-Task**				
Off-Task: No Change	2 (18%)	1 (17%)	1 (20%)	
Off-Task: Fatigue	0 (0%)	0 (0%)	0 (0%)	

Note. This table displays the on/off-task categorization of participants that completed /a-i/ assessments during /sa-ʃa/ testing. Fisher’s exact test indicated no statistically significant difference in category distribution between CNH and CHH groups (*p* = 0.11).

## Data Availability

De-identified data will be made available following institutional review and approval.

## Abbreviations

The following abbreviations are used in this manuscript:

CHH	children who are hard-of-hearing
CHT	condition head turn
CNC	could not condition
CNH	children with normal hearing
CPA	conditioned play audiometry
d′	d prime
dB	decibel
DL	datalogging
DSL	desired sensation level
EDHI	Early Hearing Detection and Intervention
F0	fundamental frequency
HA	hearing aid
Hz	Hertz
ID	identification
MEF	middle ear function
MMN	mismatch negativity
MMR	mismatch response
msec	millisecond
PTA	pure tone average
RECD	real-ear-to-coupler difference
RMSE	root mean square error
SII	speech intelligibility index
SPL	sound pressure level
UNHS	Universal Newborn Hearing Screening
VRA	visual reinforced audiometry

## Appendix A

**Table A1:** CHH participant details.

Subject	HA Manufacturer	Age at Fit (Months)	Age at ID (Days)	Better Hearing Ear	PTA	Aided SII	RMSE	DL	SII (2)	RMSE	DL (2)
14	A	2.04	48	Left	30.25	0.94	2	9.6			
15	A	2.27	13	Right	675	0.69	1.7	13.4			
16	A	2.56	21	Both	37.5	0.95	1.6	11			
17	A	1.45	8	Left	64.75	0.57	2.1	10.9			
18	A	2.73	63	Right	22.5	0.96	1	1			
19	B	4.01	44	Right	27	0.9	1.7	10.63			
20	A	2.23	17	Left	72.5	0.41	7.5	16			
21	A	2.5	34	Right	44.75	0.85	2.3	10.9			
22	A	2.23	14	Right	67.25	0.5	3.8	8.6			
23	B	3.09	54	Both	30	0.92	1.1	2.68			
24 *	A	2.07	35	Right	24.25	0.95	1.6	1.7	0.98	4.9	1.1
25 *	B	2.66	12	Both	36.5	0.9	1	7.5	0.94	7.5	
26	B	2.6	29	Left	32.5	0.94	1.7	0.12			
27	B	2.04	22	Right	21.25	0.95	8.4	0.8			
28	A	2.73	54	Right	37	0.9	4.8	7			
29	B	5.13	96	Left	47.75	0.74	3.6	5.5			
30	A	2.63	38	Both	51.25	0.78	2.2	5			
14	A	2.04	48	Left	30.25	0.94	2	9.6			
15	A	2.27	13	Right	675	0.69	1.7	13.4			
16	A	2.56	21	Both	37.5	0.95	1.6	11			
17	A	1.45	8	Left	64.75	0.57	2.1	10.9			
18	A	2.73	63	Right	22.5	0.96	1	1			
19	B	4.01	44	Right	27	0.9	1.7	10.63			
20	A	2.23	17	Left	72.5	0.41	7.5	16			

If there was a discrepancy between ears, the hearing aid related values were used from the better hearing ear’s hearing aid. Four Frequency Pure Tone Average (PTA) was averaged for the better hearing ear across 500, 1000, 2000, and 4000 Hz when thresholds were available; Hearing aid (HA); SII (Speech Intelligibility Index); Root Mean Square Error (RMSE); Datalogging (DL) * 1st set of SII/RMSE were measured using average real ear to coupler difference (RECD); 2nd set of SII /RMSE were measured using measured RECD.

**Table A2:** Participant details who completed /ba-da/ contrast.

Subject	Hearing Status	Total # of Visits (# Normal MEF)	/ba-da/ T1 Score (d′)	/ba-da/ T1 Age (Months)	/ba-da/ T2 Score (d′)	/ba-da/ T2 Age (Months)	/a-i/ Check Used	Unaided /ba-da/ Score (d′)	Unaided /ba-da/ Age (Months)	Time Between Visits (Months)
1	CNH	3 (3)	CNC	11.70	−0.11	12.29	Yes			0.59
2	CNH	2 (2)	CNC	15.74	CNC	16.19	Yes			0.45
3	CNH	2 (2)	0.88	36.83	1.47	36.83	Yes			0.00
4	CNH	1 (0) **	0.85	18.23	2.14	18.23	Yes			0.00
5	CNH	3 (3)	CNC	32.95			No			0.00
6	CNH	1 (1)	2.30	35.58			No			0.00
7	CNH	1 (1)	0.31	29.83	0.31	29.83	Yes			0.00
8	CNH	1 (1)	0.75	13.90	0.75	13.90	Yes			0.00
9	CNH	2 (2)	0.75	12.81	0.88	14.19	Yes			1.38
10	CNH	2 (0) *	CNC	24.21			No			0.00
11	CNH	1 (1)	1.74	29.37			No			0.00
12	CNH	3 (3)	CNC	28.09	−0.54	28.09	Yes			0.00
13	CNH	3 (3)	CNC	9.40	2.14	10.05	Yes			0.65
14	CHH	6 (1)	1.07	30.58	0.39	30.58	Yes	0.31	30.58	0.00
15	CHH	3 (3)	CNC	32.49	CNC	33.08	Yes			0.59
16	CHH	3 (2)	0.31	20.04	−0.08	20.04	Yes	1.24	20.04	0.00
17	CHH	4 (4)	0.39	9.92	1.74	10.45	Yes	−0.11	9.92	0.53
18	CHH	4 (2)	CNC	29.43			No	CNC	26.84	2.59
19	CHH	3 (3)	0.39	10.78			No	−0.49	10.09	0.69
20	CHH	3 (1)	0.00	34.60			No			0.00
21	CHH	1 (1)	2.14	18.13			No	−0.57	18.13	0.00
22	CHH	4 (3)						CNC	35.87	0.00
23	CHH	1 (1)	1.72	13.04			No	0.25	13.04	0.00
24	CHH	3 (3)	0.75	24.44	0.75	25.16	No	1.78	24.44	0.72
25	CHH	2 (2)	0.75	12.61	0.31	12.61	Yes	−0.07	12.61	0.00
26	CHH	2 (2)	CNC	9.40	CNC	10.12	Yes			0.72
27	CHH	2 (2)	0.79	12.45	1.47	13.86	Yes	0.97	13.86	1.41
28	CHH	3 (3)	0.75	24.38			No			0.00
29	CHH	1 (1)	1.72	12.84			No	0.18	12.84	0.00
30	CHH	2 (2)	−0.85	10.78	1.74	10.78	Yes	−0.8	10.78	0.00

NOTE: Total # of Visits represents the testing required to complete the entire protocol for all contrasts. T = time in 1 or 2 with age reported in months, Could not condition (CNC) was marked if the child could not complete the conditioning or testing phases. Middle Ear Function (MEF) was assessed by 226 Hz tympanometry and was considered abnormal if either ear presented with and abnormal tympanogram. * Significant distortion product otoacoustic emissions (per protocol only assessed among CNH) were present despite abnormal tympanometry indicating sound transmission. ** MEF and OAEs were measured for Subject 4 at the end of the testing session. Results were included due to participant meeting criterion prior to measurement.

**Table A3:** Participant details who completed /sa-ʃa/ contrast.

Subject	Hearing Status	Total # of Visits (# Normal MEF)	/sa-ʃa/ T1 Score (d′)	/sa-ʃa/ T1 Age (Months)	/sa-ʃa/ T2 Score (d′)	/sa-ʃa/ T2 Age (Months)	/a-i/ Check Used	Unaided /sa-ʃa/ Score (d′)	Unaided /sa-ʃa/ Age (Months)	Time Between Visits (Months)
1	CNH	3 (3)	1.71	11.7	1.78	12.29	No			0.59
2	CNH	2 (2)	1.72	15.74			No			0.00
3	CNH	2 (2)	1.72	36.83			No			0.00
4	CNH	1 (0) **	0.85	18.23	1.72	18.23	Yes			0.00
5	CNH	3 (3)	CNC	31.34	CNC	32.03	Yes			0.69
6	CNH	1 (1)	1.82	35.58			No			0.00
7	CNH	1 (1)	1.72	29.83			No			0.00
8	CNH	1 (1)	1.07	13.90	2.60	13.90	Yes			0.00
9	CNH	2 (2)	CNC	14.19	−0.5	14.19	Yes			0.00
10	CNH	2 (0) *	0.18	23.98			No			0.00
11	CNH	1 (1)	2.22	29.37			No			0.00
12	CNH	3 (3)	CNC	24.84	1.24	25.79	Yes			0.95
13	CNH	3 (3)	1.07	10.48	2.62	10.48	Yes			0.00
14	CHH	6 (1)	1.15	30.06	CNC	30.58	Yes	1.39	30.06	0.52
15	CHH	3 (3)	1.47	32.49			No			0.00
16	CHH	3 (2)	1.24	18.63			No	0.57	18.63	0.00
17	CHH	4 (4)	0.79	10.45			No	CNC	10.35	0.10
18	CHH	4 (2)	2.14	29.43			No	1.47	29.43	0.00
19	CHH	3 (3)	−0.14	11.14			No	CNC	10.09	1.05
20	CHH	3 (1)	1.38	34.53			No			0.00
21	CHH	1 (1)	3	18.13			No	1.47	18.13	0.00
22	CHH	4 (3)	1.39	37.25			No	0.75	37.25	0.00
23	CHH	1 (1)	1.24	13.04	0.75	13.04	Yes	0.75	13.04	0.00
24	CHH	3 (3)	CNC	25.15	CNC	26.08	No	0.88	24.44	1.64
25	CHH	2 (2)	0.75	12.61	1.33	13.76	No	−0.08	12.61	1.15
26	CHH	2 (2)	0.88	10.12	1.07	10.12	Yes			0.00
27	CHH	2 (2)	1.24	12.45			No	1.33	13.86	1.41
28	CHH	3 (3)	CNC	22.07	0.57	23.49	Yes			1.42
29	CHH	1 (1)	0.58	12.84			No	1.33	12.84	0.00
30	CHH	2 (2)	−0.49	11.14	0.79	11.14	Yes	0.39	11.14	0.00

OTE: Total # of Visits represents the testing required to complete the entire protocol for all contrasts. T = time in 1 or 2 with age reported in months, Could not condition (CNC) was marked if the child could not complete the conditioning or testing phases. Middle Ear Function (MEF) was assessed by 226 Hz tympanometry and was considered abnormal if either ear presented with an abnormal tympanogram. * Significant distortion product otoacoustic emissions (per protocol only assessed among CNH) were present despite abnormal tympanometry indicating sound transmission. ** MEF and OAEs were measured for Subject 4 at the end of the testing session. Results were included due to participant meeting criterion prior to measurement.
